# Supplementary material for: “Post-GDM support would be really good for mothers”: A qualitative interview study exploring how to support a healthy diet and physical activity after gestational diabetes
Source: PLoS One. 2022 Jan 21;17(1):e0262852. doi: 10.1371/journal.pone.0262852 (PMC8782419; doi:10.1371/journal.pone.0262852)
Supplement: S1 Table — (PDF) [file pone.0262852.s001.pdf]

“Post-GDM support would be really good for mothers”: a qualitative interview study exploring how to support a healthy diet and physical activity after gestational diabetes

R A Dennison, S J Griffin, J A Usher-Smith, R A Fox, C E Aiken, C L Meek

Table S1. DAiSIeS interview schedule.

Introduction
- Introduce researcher and the purpose of the interview<br>- Explain the interview procedure<br>- Informed consent<br>- Begin recording
As I said, I am particularly interested in post-pregnancy, but perhaps you could start by telling me a little about what your GDM pregnancy was like for you?
Understand current lifestyle
To help us understand any lasting impact GDM might have had, please tell me a bit about your current diet.
What was your diet like before your gestational diabetes pregnancy? [How] do you think this has changed? What helped you make these changes?
Please tell me a bit about anything that you do to stay active.
Did you do any exercise before your gestational diabetes pregnancy? [How] do you think this has changed?
To summarise, do you think GDM has had an impact on you, making any lasting changes?
[If any,] what other changes to your diet/exercise would you like to make? Why do you say this?
Ideal lifestyle intervention
What would help you most to have a healthier lifestyle/to make the changes we’ve spoken about?
Introduce suggestion cards 1–10. Are there any that would be beneficial to you? Any that wouldn’t be? Anything that you would add?
Is there anything else that you would like to add about diet and exercise?
Understand current screening behaviour
Have you had a test for diabetes since your pregnancy? What made you go/what prevented you from going?
How do you feel about having regular diabetes tests in the future?
Ideal screening intervention
What would help you most to attend diabetes testing? (At 6 weeks postpartum and annually)
Introduce suggestion cards 11–20. Are there any that would be beneficial to you? Any that wouldn’t be? Anything that you would add?
Close

- Today I wanted to talk to you about diet, exercise and screening for T2D after GDM, and what might help with this. Is there anything else that you would like to discuss or ask?<br>- Complete questionnaire<br>- End<br><br>*Suggestion cards*

1. More information about the impact of healthy diet/exercise on your diabetes risk	2. More information about the impact of healthy diet/exercise on your wider health (e.g. stress, weight)
3. More information about the impact of healthy diet/exercise on your family	4. Suggested ways for your children and wider family to be healthier
5. Help for you to exercise with others	6. Advice about how to have a healthy diet (food shopping, cooking, healthy substitutions, etc.)
7. Advice about how to exercise with a busy schedule (e.g. around the home)	8. Advice about how to keep going with healthy changes to your diet/exercise
9. Advice about saving money and healthy diet/exercise	10. Monitoring your progress
11. Doctors talking more about postpartum tests while you were pregnant	12. Invitations and reminders for tests
13. Your GP knowing more about your pregnancy	14. More opportunities to understand gestational diabetes
15. More child-friendly clinics/waiting rooms	16. Being able to get tested at a place of your choice
17. Shorter, more pleasant tests	18. Combining glucose testing with other appointments
19. A better understanding of the purpose of glucose testing	20. Not being able to monitor your blood sugar yourself
