# Supplementary material for: “Post-GDM support would be really good for mothers”: A qualitative interview study exploring how to support a healthy diet and physical activity after gestational diabetes
Source: PLoS One. 2022 Jan 21;17(1):e0262852. doi: 10.1371/journal.pone.0262852 (PMC8782419; doi:10.1371/journal.pone.0262852)
Supplement: S2 Table — (PDF) [file pone.0262852.s002.pdf]

“Post-GDM support would be really good for mothers”: a qualitative interview study exploring how to support a healthy diet and physical activity after gestational diabetes

R A Dennison, S J Griffin, J A Usher-Smith, R A Fox, C E Aiken, C L Meek

Table S2. Thematic framework used to analyse the DAiSIeS interviews.

1. **Diet and exercise**
    a. Diet
        i. General comments
        ii. Prompted suggestion card #6 Advice about how to have a healthy diet
        iii. Unprompted suggestions
    b. Exercise
        i. General comments
        ii. Prompted #5 Help for you to exercise with others
        iii. Prompted #7 Advice about how to exercise with a busy schedule
        iv. Unprompted suggestions
    c. Information and understanding
        i. General comments
        ii. Prompted #1 More information about the impact of healthy diet/exercise on your diabetes risk
        iii. Prompted #2 More information about the impact of healthy diet/exercise on your wider health
        iv. Unprompted suggestions
    d. Family
        i. General comments
        ii. Prompted #3 More information about the impact of healthy diet/exercise on your family
        iii. Prompted #4 Suggested ways for your children and wider family to be healthier
        iv. Unprompted suggestions
    e. Money
        i. General comments
        ii. Prompted #9 Advice about saving money and healthy diet/exercise
        iii. Unprompted suggestions
    f. Sustainability
        i. General comments
        ii. Prompted #8 Advice about how to keep going with healthy changes to your diet/exercise
        iii. Unprompted suggestions
    g. Monitoring
        i. General comments
        ii. Prompted #10 Monitoring your progress
        iii. Unprompted suggestions

2. **Diabetes screening**

    Reported separately

3. Other

i. Pregnancy
ii. Mode of delivery
iii. Source and who
iv. When
v. Suggested content of a postpartum appointment
vi. Sensitivity and non-judgemental attitude
vii. General postpartum experience
viii. Other

Framework analysis charts were drawn for each second-level item (indicated by letters a–g). #N indicates the number of the suggestion card referred to.
