# Supplementary material for: “Post-GDM support would be really good for mothers”: A qualitative interview study exploring how to support a healthy diet and physical activity after gestational diabetes
Source: PLoS One. 2022 Jan 21;17(1):e0262852. doi: 10.1371/journal.pone.0262852 (PMC8782419; doi:10.1371/journal.pone.0262852)
Supplement: S3 Table — Agreement was based on the authors’ interpretation of their responses. Not all participants were shown each card, and some did not comment or agreement was unclear. Dark green: strongly agree; green: agree; red: disagree; dark red: strongly disagree; grey: not shown or agreement is unclear. A: overall agreement; M: overall mixed response; D: overall disagreement. (PDF) [file pone.0262852.s003.pdf]

"Post-GDM support would be really good for mothers": a qualitative interview study exploring how to support a healthy diet and physical activity after gestational diabetes

R A Dennison, S J Griffin, J A Usher-Smith, R A Fox, C E Aiken, C L Meek

Table S3. DAiSIeS participants' agreement with whether the suggestion cards will support healthy diet and physical activity.

	Suggestion card										
**Participant**	1. More information about diet/exercise on T2D risk	2. More information about diet/exercise on wider health	3. More information about diet/exercise on family	4. Suggestions for healthy families	5. Support to exercise with others	6. Advice about how to eat healthily	7. Advice about exercising with a busy schedule	8. Advice about sustaining changes	9. Advice about saving money	10. Advice about monitoring progress	**Overall response**
**P1**	×	×	×	×	×	✓	✓	✓	✓	✓	M
**P2**	×	×	✓	✓	✓	✓	✓	✓	×	✓	A
**P3**	×	×	×	×	×	×	×	×	×	×	D
**P4**	✓	✓	✓	✓	✓	✓	✓	✓	✓	✓	A
**P5**	✓	×	×	×	×	✓	×	✓	×	✓	M
**P6**	✓	✓	✓	✓	×	×	✓	✓	✓	✓	A
**P7**	✓			✓	✓	✓	✓	✓	×	✓	A
**P8**	✓	✓	✓	✓	✓	✓	✓	✓	✓	✓	A
**P9**	✓	✓	✓	✓	✓	×	✓	✓	✓	✓	A
**P10**	✓	✓	✓	✓	✓	✓	✓	✓	✓	✓	A
**P11**	✓	✓	✓	✓	×	✓	✓	✓	✓	✓	A
**P12**	✓	×	×	×	✓	✓	✓	×	✓		M
**P13**	✓	✓	✓	✓	✓	✓	✓	✓	✓	✓	A
**P14**	×	✓	✓	✓	✓	✓	✓	✓	✓	✓	A
**P15**	✓		×	×	✓	✓	✓	✓	✓	✓	A
**P16**	✓	✓		✓	✓	✓	✓		✓	✓	A
**P17**	✓	×	×	×	✓	×	✓	✓	×	✓	M
**P18**	✓	✓		✓		✓				✓	A
**P19**	✓	✓	×	×	×	✓	✓	✓	✓	✓	A
**P20**	×	×	×	×	✓	✓	✓	×	×	×	D
Overall response	A	M	M	M	A	A	A	A	M	A	

Agreement was based on the authors' interpretation of their responses. Not all participants were shown each card, and some did not comment or agreement was unclear.

Dark green: strongly agree; green: agree; red: disagree; dark red: strongly disagree; grey: not shown or agreement is unclear.

A: overall agreement; M: overall mixed response; D: overall disagreement.
