# Supplementary material for: “Post-GDM support would be really good for mothers”: A qualitative interview study exploring how to support a healthy diet and physical activity after gestational diabetes
Source: PLoS One. 2022 Jan 21;17(1):e0262852. doi: 10.1371/journal.pone.0262852 (PMC8782419; doi:10.1371/journal.pone.0262852)
Supplement: Attachment — Submitted filename: Manuscript Number PONE-D-21-01829.docx [file pone.0262852.s004.docx]

**TITLE: “POST-GDM SUPPORT WOULD BE REALLY GOOD FOR MOTHERS”: A QUALITATIVE INTERVIEW STUDY EXPLORING HOW TO SUPPORT A HEALTHY DIET AND PHYSICAL ACTIVITY AFTER GESTATIONAL DIABETES**

| **Line** | **Item** |
| --- | --- |
| **Title** | **There is** a **tautology in the title (A qualitative interview study exploring how to support a healthy diet and physical activity after gestational diabetes). The second part of the title is more suitable and straightforward than (“post-GDM support would be really good for mothers”: a qualitative interview study exploring how to support a healthy diet and physical activity after gestational diabetes). The title's preamble and the title itself are the same, so the title should be reframed to make more coincide.** |
| **Abstract** |  |
| 24 | developing type 2 diabetes mellitus (T2DM). Be specific about the type of diabetes being discussed |
| 26/27 | The research sought to explore women’s views on suggested …  maybe more appropriate |
| 28/29 | Semi-structured interviews with 20 participants in Cambridgeshire, UK at 3–48 months after GDM were conducted |
| 29/30 | ~~Their~~ current diet and physical activity, intentions for any changes, and views on potential interventions to help manage T2DM risk through these behaviours were discussed. |
| 31/32 | ~~We based~~ the interview schedule, suggested interventions, and thematic framework was based on a recent systematic review |
| 53/54 | which percentage is the emphasis on? The bracket? |
| **Introduction** | |
| 65/73 | The context in this session is in isolation |
|  |  |
| **Participants and Methods** | |
|  | **Recruitment:** this session sounds more like a narration due to the use of "we." |
| 118-120 | **Interview process: informed consent implies** “they understood the purpose and procedure of the study and that they could stop the interview at any point, and were happy for it to be audio-recorded”. Hence, this addition may not be necessary |
| 89/90 | Why is the reference? |
| **Results** | |
|  | Well presented |
| **Conclusion** | |
| 406 | The range of formats could be mentioned |
| References | |
|  | Well presented |

1. No
