# Supplementary material for: “Post-GDM support would be really good for mothers”: A qualitative interview study exploring how to support a healthy diet and physical activity after gestational diabetes
Source: PLoS One. 2022 Jan 21;17(1):e0262852. doi: 10.1371/journal.pone.0262852 (PMC8782419; doi:10.1371/journal.pone.0262852)
Supplement: Attachment — Submitted filename: Response to reviewers_PONE-D-21-01829R1.docx [file pone.0262852.s005.docx]

**Reviewers' comments**

**Reviewer's Responses to Questions**

1. If the authors have adequately addressed your comments raised in a previous round of review and you feel that this manuscript is now acceptable for publication, you may indicate that here to bypass the “Comments to the Author” section, enter your conflict of interest statement in the “Confidential to Editor” section, and submit your "Accept" recommendation.

Reviewer #3: (No Response)

Reviewer #4: (No Response)

Reviewer #5: (No Response)

Reviewer #6: All comments have been addressed

________________________________________

2. Is the manuscript technically sound, and do the data support the conclusions?

The manuscript must describe a technically sound piece of scientific research with data that supports the conclusions. Experiments must have been conducted rigorously, with appropriate controls, replication, and sample sizes. The conclusions must be drawn appropriately based on the data presented.

Reviewer #3: Partly

Reviewer #4: Partly

Reviewer #5: Partly

Reviewer #6: Yes

________________________________________

3. Has the statistical analysis been performed appropriately and rigorously?

Reviewer #3: Yes

Reviewer #4: N/A

Reviewer #5: N/A

Reviewer #6: Yes

________________________________________

4. Have the authors made all data underlying the findings in their manuscript fully available?

The PLOS Data policy requires authors to make all data underlying the findings described in their manuscript fully available without restriction, with rare exception (please refer to the Data Availability Statement in the manuscript PDF file). The data should be provided as part of the manuscript or its supporting information, or deposited to a public repository. For example, in addition to summary statistics, the data points behind means, medians and variance measures should be available. If there are restrictions on publicly sharing data—e.g. participant privacy or use of data from a third party—those must be specified.

Reviewer #3: Yes

Reviewer #4: No

Reviewer #5: Yes

Reviewer #6: Yes

________________________________________

5. Is the manuscript presented in an intelligible fashion and written in standard English?

PLOS ONE does not copyedit accepted manuscripts, so the language in submitted articles must be clear, correct, and unambiguous. Any typographical or grammatical errors should be corrected at revision, so please note any specific errors here.

Reviewer #3: Yes

Reviewer #4: Yes

Reviewer #5: Yes

Reviewer #6: Yes

________________________________________

6. Review Comments to the Author

**Reviewer #3**

FPG test ?? in Figure 2. Please provide the full form of FPG test before providing the abbreviated form.

RESPONSE: We have added the relevant abbreviations to the caption of the figures.

**Reviewer #4**

Thank you very much for having me to review the work entitled “Post-GDM support would be really good for mothers”: a qualitative interview study exploring how to support a healthy diet and physical activity after gestational diabetes.

The study aims to explore women’s views on suggested practical approaches to achieve and maintain a healthy diet and physical activity to reduce T2D risk. Please consider the following comments to improve the manuscript.

1. Abstract

Line 33 – Line 35: Suggest to include the percentages of “a third” participants for “transformative”, “beneficial” and “did not want additional support”.

Line 35: Suggest to put the percentage after “the majority”.

Line 38: Suggest to put the percentage after “four”.

RESPONSE: Thank you for reviewing our manuscript and for your suggestions to improve it.

As we explain in detail below for the results section (page 13 of this response letter), this is primarily a qualitative analysis. While semi-quantification can be useful to convey the relative importance to the reader (particularly in the abstract where the opportunity for a detailed description is limited), this is not the emphasis of the analysis. Consequently, it would not be appropriate to add percentages into the abstract.

2. Introduction

Line 55 - Line 56: Since you mentioned “These sites were chosen to provide socioeconomic and ethnic diversity, and represent views from those attending both secondary and tertiary centres offering GDM/ and obstetric care” in line 80 to line 81 (page 4), hence, it is better to specify the prevalence of GDM of different ethnicities (Chinese, Indian, Japanese) instead of putting “as compare to other populations”.

RESPONSE: We have added more information about the epidemiology of GDM in the introduction, particularly considering prevalence according to ethnicity and geographical region. The new text is highlighted in bold below, and line numbers refer to those in the revised manuscript with tracked changes.

“An estimated 17.8 million pregnancies resulting in live births were affected by gestational diabetes mellitus (GDM) worldwide in 2015 [1]. **Estimates of prevalence vary greatly within and between regions and countries: the Middle East and North Africa have the highest prevalence at a median 12.9% of pregnancies affected (range 8.4 to 24.5%) and Europe has the lowest prevalence at 5.8% (range 1.8 to 22.3%) [2]; in Eastern and South-Eastern Asia, prevalence is 10.1% (95% confidence interval 6.5% to 15.7%) [3].** Approximately 5% of UK pregnancies were affected in 2015 [4]. **Compared to women of an ethnicity associated with a high risk of GDM who are born in a Western country, many women who migrated from their native country to a Western country have higher rates of GDM [5].**” (lines 48-56).

Additionally, we have added another statement describing the incidence of T2D after GDM:

“T2DM risk factors including high body mass index (BMI) and ethnicity further increase T2DM risk in women who have had GDM: development of T2DM is … 57% (95% confidence interval 39–70%) lower in White European populations compared to other populations (adjusting for ethnicity and follow-up) [7]. **Women from Asia were found to have the highest incidence rate of T2DM after GDM at 46 cases per 1,000 person-years [8].**” (lines 61-66).

Line 57: Please specify the type of risk for “glucose tolerance requiring treatment with insulin have been found to further increase risk”. It this refer to GDM risk or T2D risk?

RESPONSE: We have clarified that we are referring to risk of T2D in this statement:

“Factors such as poorer pregnancy glucose tolerance requiring treatment with insulin have been found to further increase risk **of T2DM** [9].” (lines 66-68).

Line 64: Please specify the activities of behaviour change intervention. For example, the type of intervention done by previous researchers to give a better picture for the readers. Support your sentence with reference.

RESPONSE: We have reported more details on previous behaviour change interventions to prevent T2D after GDM:

“Existing behaviour change interventions have **focused on promoting physical activity and a healthy diet, while others have supported breastfeeding after GDM [13]. Intervention modes include group, individual and remote interventions, or a combination of approaches [13].** Positive effects on preventing T2DM progression are frequently **observed but can be** limited due to poor engagement, **particularly in intensive interventions like the US Diabetes Prevention Programme,** in this population [13–16].” (lines 74-79).

Line 65: Please specify the type of population. Is this referring to women with GDM history or without GDM history? Because GDM history may influence the effectiveness of the behavioural change intervention.

RESPONSE: Throughout this paper, the population of interest is women with a history of GDM. We are explicit if this is not the case (in the discussion). We have reminded the reader of this, such as in lines 71-74:

“Nevertheless, most women **who have had GDM** do not attempt or sustain changes to reduce modifiable risk factors but maintain lifestyles that increase their diabetes risk, and many show discrepancy between T2DM risk perception and behaviour [12].”

Line 66 - Line 67: Please provide a reference for “we found that women who had had GDM identified themselves primarily as mothers who prioritised their family above themselves”.

RESPONSE: We have added a reference for this sentence.

Line 71: Considering a small sample size in the present study, please provide the sample size for Reference 13 and Reference 14. This is to give an overall picture for the readers to understand the common sample size being used in qualitative research.

Line 71: Please provide brief details for Reference 13 and Reference 14. What are the previous activities conducted by the researchers?

RESPONSE: We have added details about Lie *et al.* 2013 and Parsons *et al.* 2014 (now references 18 and 19):

“Only one of the 21 included studies was set in the UK **(interviews with 35 women in total to explore influences on postpartum health behaviours and the feasibility of diabetes prevention intervention [18])** and we are aware of only one other UK study that has been completed more recently **(interviews or focus groups with 50 women in total also to explore influences on postpartum health behaviours and preferences for lifestyle support [19])**.” (lines 87-91).

We would like to remind the reviewer that while our sample size of 20 participants was larger than many semi-structured interview studies, a larger sample size does not indicate a better qualitative study.^1^ The qualitative systematic review that informed this interview study included 12 studies using face-to-face interviews.^2^ Only three of those had a sample size >20 participants (Lie *et al.* 2013 was the largest with 35 participants) and median sample size was 16 participants.

Line 72: Please mention the research gap before your study adjective.

RESPONSE: We have expanded on the contribution of this study to the literature:

“**There is therefore a gap in recent literature in the UK population surrounding the acceptability of recommendations for intervention after GDM in a real-life context.** In this study, **we sought to address this gap by exploring** the views of women with a history of GDM on possible interventions to support healthy diet and physical activity to reduce diabetes risk, in addition to participants’ own suggestions. We aimed to identify the most promising interventions for future development.” (lines 92-97).

Line 74: Is this objective “We aimed to identify the most promising interventions for future development” reflect in your findings? If not, please remove this sentence.

RESPONSE: This objective is reflected in the finding that a range of interventions are needed and acceptable – there was not just one that the participants supported. We have clarified this in the conclusions of both the abstract and main text by saying:

“Many women would appreciate more support to reduce their T2DM risk after GDM and believe that **a variety of** interventions to integrate changes into their daily lives would help them to sustain healthier lifestyles.” (lines 44-46).

“Many women wanted more support to sustain healthy lifestyles to reduce their T2DM risk after a GDM-affected pregnancy. **We identified a broad range of interventions that could offer this support.**” *Then summarise what these interventions are* (lines 486-487).

3. Recruitment

Line 86: Please provide the response rate of this study. Out of the total contacted participants, how many of them agreed or rejected to participate in the present study.

RESPONSE: As described at the end of this paragraph (now lines 110-119), we interviewed all participants who wanted to take part. Those who recruited the participants have added that they invited several hundred women with GDM diagnosed within the last approximately 5 years, and also spoke directly to some women in hospital clinics. Uptake was around 50% for women approached directly and around 5% for women approached by means of a letter. We did not record the final number invited directly so are unable to present the exact response rate. Similarly, we do not know how many people saw the awareness posters that we displayed in clinics. We have added this additional estimate of uptake in the methods:

“We interviewed all **20** participants who wanted to take part. Although we did not record the final number invited, **uptake was estimated to be around 50% for women spoken to directly and around 5% for women who were sent a letter or email only**.” (lines 118-120).

However, we do know that the participants in this study were representative of the women diagnosed with GDM accessing care at these hospitals. Meek *et al.* 2021^3^ (Table 2) and Lachmann *et al.* 2020^4^ (Table 1) report the age, parity, and ethnicity of women accessing care at the Rosie Hospital and Peterborough Hospital (OPHELIA cohort). In fact, we interviewed a slightly lower proportion of White women than seen in the clinic normally (80–90% in clinic). There is little previous data about occupation or education, but the authors who work at these clinics consider that this is also representative of their patients. Common with other studies, it is often difficult to obtain representation from women with active mental health concerns, challenging social circumstances, or who speak no English. We addressed this by approaching women from these groups directly, but cannot exclude the possibility of under-representation here.^4^

We have therefore made this more explicit and included additional information in the strengths and limitations section of the discussion:

“This [the need for postpartum support] may have been influenced by recruitment bias, with more health-conscious women or those in need of particular support more likely to engage in the study. **We were also unable to capture the number or any characteristics about the women who were aware of the study (through seeing the posters displayed at GDM clinics or being invited specifically) and chose not to take part. However, the participants’ characteristics were comparable to those of the women attending these clinics [31,32].**” (lines 431-436)

We would like to highlight that although a limitation, this is not a significant issue because, as discussed in our previous response letter, the aim of qualitative research is not necessarily to be representative of the wider population but to grasp an in-depth understanding/interpretation of the views of a particular group of people.^1,5–7^

Line 87 – Line 89: These sentences are not strong enough to support your final sample size (n=20). Since you mentioned “to reach data saturation”, have you conducted data saturation assessment in your study? Please explain how you assess the data saturation.

Line 88 – Line 89: What do you mean by “this study had a broad aim sparse sample specificity but used purposive sampling and was a cross-case analysis”? This sentence is unclear and needs further improvement.

Line 90: What do you mean by “the interviews were structured around pre-defined recommendation”? Please provide more details and also a reference for this statement.

RESPONSE: Reference 20, Malterud *et al.* 2016, presents ‘the concept “information power” to guide adequate sample size for qualitative studies’. They ‘identified five items that along different dimensions have an impact on the information power of the sample: (a) study aim, (b) sample specificity, (c) use of established theory, (d) quality of dialogue, and (e) analysis strategy.’ ‘A study will need the least amount of participants when the study aim is narrow, if the combination of participants is highly specific for the study aim, if it is supported by established theory, if the interview dialogue is strong, and if the analysis includes longitudinal in-depth exploration of narratives or discourse details’ and vice versa.

We have revised this paragraph to more explicitly explain how we used information power to determine the approximate sample size, and clarified the other points raised:

“We planned to interview approximately 20 women in order to reach data saturation, **a comparatively large sample size** based on the relatively low information power anticipated [20]. This was because this study had a broad aim **(to explore the participants’ views on potential interventions) and the sample was not very specific (participants had a recent history of GDM but no criteria relating to lifestyle behaviours).** **One the other hand,** the interview schedule **and framework analysis** were structured around the recommendations **made in our systematic review [17], which increases the information power. As widely recommended for data saturation [21],** we finished recruitment after several interviews did not lead to novel findings.” (lines 110-118).

Line 90 - Line 91: Is Reference 16 correctly cited?

RESPONSE: This reference, now reference number 21, summarises several debates concerning what sample size is the right size for qualitative studies, suggesting that ‘Saturation is defined by many as the point at which the data collection process no longer offers any new or relevant data.’ (Dworkin 2012, page 1). We have revised this sentence to read:

“**As widely recommended for data saturation [21],** we finished recruitment after several interviews did not lead to novel findings.” (lines 116-118).

Line 91 – Line 92: How many participants you have interviewed in your study? Do you include data of all participants that have completed the interview or only part of them? Please provide the response rate.

RESPONSE: We have clarified that “We interviewed all **20** participants who wanted to take part…” (line 118). All of the interviews were analysed and included in this study.

4. Inclusion criteria

Line 98: Do you conduct GDM screening in your study? Or do you obtain GDM data from the participants’ medical history?

RESPONSE: As stated under the Recruitment section, “Research midwives identified eligible participants from medical records, and sent them a postal or email invitation and participant information sheet describing the study.” (lines 105-107). This was performed using hospital electronic medical record systems.

Line 110 – Line 113: Please mentioned how many women you have excluded from the study.

RESPONSE: We do not have data on the number of who were not considered for the study due to adverse pregnancy outcomes (such as stillbirth, neonatal death or major congenital anomaly), participation in a pregnancy or GDM-related lifestyle intervention (very few during the study period) or were considered unsuitable for any other reason (lines 118-120 and 138-141) at the discretion of the midwives who had access to their medical records. However, we designed the study to include virtually all women with a history of GDM so we expect very few women were ineligible.

5. Interview process

Do you conduct any pre-test prior to the interview? Modification of interview guide and suggestion cards are required before you conduct the actual interview. If yes, how many attended the pre-test and what modifications you have done?

RESPONSE: Written feedback from the patient and public involvement group and feedback from pilot interviews was incorporated into the final version of the interview guide and suggestion cards. As is normal practice, these were refined after reflection on the first interviews. We have added a description of the changes made to the interview schedule:

“**We conducted three pilot interviews and collected** written feedback from our patient and public involvement group, composed of mothers with GDM. **This feedback** was incorporated into the final version, which was refined after reflection on the first interviews. **This involved changing the first question of the interview schedule from asking about their current diet (which we expected would be an easy discussion topic) to asking them to describe “what your GDM pregnancy was like for you”. Although this was not the focus of the interview and sometimes brought up upsetting accounts, the participants appreciated being able to tell their ‘story’ and it proved to be useful context for the remainder of the interview. Additionally, more signposting was incorporated into the interview such as “To help us understand any lasting impact GDM might have had…” or “Before we talk about exercise, I’d like to ask you about your diet”.**” (lines 153-163).

Line 122 - Line 123: Based on Table S2, it seems like No 11 to 20 in Table S1 are not including in your study. Please clarify the number of suggestion cards in your study. Please mention how many suggestion cards you have included in the final version.

RESPONSE: The aim of the *entire* interview was to understand how to support women who have had GDM to prevent or delay progression to T2D. Having a healthy lifestyle (healthy diet and physical activity) and attendance at diabetes screening are the two key, long-term recommended behaviours.

The interview consisted of two parts – firstly, a discussion about diet and physical activity; secondly, a discussion about screening for T2D after GDM. The diet and physical activity part was informed by our 2019 paper,^2^ which had 20 recommendations for promoting healthy behaviour, which we adapted into 10 suggestion cards (Fig 1). The screening part was informed by our 2020 paper,^8^ which had 10 recommendations for promoting healthy behaviour, which we adapted into 10 suggestion cards.

The paper focussing on the screening part of the interviews has already been published.^9^

For completeness, we have reported the full interview schedule in Table S2.

We have clarified this in the text by saying:

“Our previous systematic review informed the interview guide and suggestion cards, which were adapted from 20 recommendations (Fig 1 and S1 Table) [17]. **The first part of the interview focused on diet and physical activity while the second part focused on screening for T2DM (reported separately [23]); ten suggestion cards were used as prompts in each part.**” (lines 150-153).

Line 132: Please elaborate type of format for “what format might be most effective”.

RESPONSE: We have clarified what we mean by format by giving examples:

“We discussed whether any support for these behaviours would be helpful, and what format might be most effective **(such as online, face-to-face with clinicians, peer support groups, etc.)**.” (lines 169-171).

Line 133: Please mention how many suggestion cards you have shown to your participants.

RESPONSE: We have added that there were ten cards:

“Participants were asked about their own ideas first, then to comment on the **ten** suggestion cards provided by the interviewer…” (lines 171-172).

Line 137: “These questions were then repeated for attending diabetes screening (reported separately)”: When did you conduct the diabetes screening? On the same day of the semi-structured interview? Do you conduct the diabetes screening on your own? Do you include the feedbacks of participants in your manuscript? Please provide the feedbacks of participants provided in the diabetes screening in the results or appendix.

RESPONSE: As clarified above, the second part of the interview aimed to elicit how to support women to attend diabetes screening after GDM (e.g. remove unnecessary barriers), which is recommended in international guidelines. This is clear from Table S1, which reports the whole interview schedule. We have also revised the sentence noted above to say:

“**We then discussed how to facilitate attendance at diabetes screening after GDM in the second part of the interview [23].**” (lines 180-181).

Line 137 - Line 138: Do you collect social-demographic data or demographic data? Please specify the type of information you have collected for demographics (ethnicity, age, occupation and etc).

RESPONSE: We have said, “Finally, demographic information **(including age band, ethnic group, employment status and pregnancy history)** and interview feedback were collected through a short questionnaire.” (lines 182-184).

Line 138: Please write the full term of “RD” instead of its acronym.

RESPONSE: We have replaced these initials with “the first author” or “the interviewer” throughout.

6. Analysis

Line 142: Please provide brief details of the framework approach.

RESPONSE: The remainder of this paragraph provides details of the framework approach, in line with the methodological publications and relevant reporting guidelines. Additionally, the framework is included in Table S2. We have added more details to this as follows:

“We used a framework approach to analyse the interviews [25], with the aid of NVivo 12 (QSR International Pty Ltd; version 12; released 2018):

1. **Familiarisation:** We familiarised ourselves with **the data by listening to** the recordings **and reading the** transcripts and field notes, **and making notes about important concepts**.
2. **Identifying a thematic framework: We** developed a thematic framework that was based on the suggestion cards then refined **it** **as required upon analysing the interviews by incorporating additional repeating concepts (such as communication requires a positive, non-judgemental tone).** We distinguished between suggestions initiated by participants and responses to the suggestion cards in the analysis. The final codebook for the framework is reported in S2 Table.
3. **Indexing:** Next, we coded each transcript **according to the thematic framework**.
4. **Charting: We** drew charts to summarise **what each participant said in relation to each part of the thematic framework. One row was used for each participant interviewed, and one column was used for each code within the framework.**
5. **Mapping and interpretation:** We carefully studied the charts for repeating or **characteristic** ideas to describe and explain the phenomena observed. **Where differences and deviant cases were observed, we attempted to understand the ways in which they different and why, according to the information the participants provided.**

The first author coded all of the transcripts and developed the charts, and another author coded and charted four transcripts to ensure **general** agreement **with the coding strategy and discuss alternative explanations.** The other authors read some or all of the transcripts and charts in order to support interpretation. During these discussions, we considered the authors’ clinical (obstetrics, diabetes and general practice) and non-clinical backgrounds **and made notes to record the analytical and interpretational decisions**.” (lines 189-214).

Line 143: Please provide the version number and manufacturer details of NVivo 12.

RESPONSE: We have updated this to NVivo 12 **(QSR International Pty Ltd; version 12; released 2018)** (lines 189-190).

Line 143 – Line 144: “…and developed a thematic framework”. Please attach the thematic framework to the manuscript.

RESPONSE: The framework is included in Table S2.

Line 144 – Line 145: Do you refine your thematic framework based on the first few interviews? Does the thematic framework represent the views of all participated participants?

RESPONSE: As described above, we have provided additional details on the analysis, including how we refined the thematic framework:

“**2. Identifying a thematic framework: We** developed a thematic framework that was based on the suggestion cards then refined **it** **as required upon analysing the interviews by incorporating additional repeating concepts (such as communication requires a positive, non-judgemental tone).** We distinguished between suggestions initiated by participants and responses to the suggestion cards in the analysis. The final codebook for the framework is reported in S2 Table.” (lines 193-198).

The thematic framework is designed to characterise the views of all participated participants.

Line 147 – Line 148: Please write the full term of “RD” and “RF” instead of their acronym.

RESPONSE: We have replaced these initials with “the first author”, “the interviewer” or “another author”, as appropriate, throughout.

Line 148: “… and charted four transcripts to ensure agreement”. Please explain how to assess agreement in your study. Do you have any references for the classification?

RESPONSE: Discussions about coding and charting were qualitative in nature. As explained by Barbour 2001, page 2, ‘the degree of concordance between researchers is not really important; what is ultimately of value is the content of disagreements and the insights that discussion can provide for refining coding frames. The greatest potential of multiple coding lies in its capacity to furnish alternative interpretations…’.^10^ We have therefore explained that:

“The first author coded all of the transcripts and developed the charts, and another author coded and charted four transcripts to ensure **general** agreement **with the coding strategy and discuss alternative explanations**.” (lines 209-211).

7. Results

Table 1: Please provide mean years of postpartum.

RESPONSE: The participants were asked to indicate their age band in the demographic questionnaire at the end of the interview (26–30 years, 31–35 years, 36–40 years, ≥41 years), as we have reported in Table 1. We did not collect data on time since pregnancy; all participants were between 12 weeks and four years postpartum at the time of the interview according to the eligibility criteria (lines 121-122).

Table 1: Since this is a descriptive analysis, it will be good to present the number of participants for each ethnicity (Chinese, Japanese, Indian).

RESPONSE: We have added additional ethnic groups, using the options used in surveys such as the UK census and PAMCo, to Table 1.

Table 1: Please check the distribution of participants of employment and maternity leave as their final number is more than 20.

Table 1: Please add the number of respondents who did not live with their partner.

Table 1: “……. All pregnancies affected by GDM”: Only 13 participants? Based on your inclusion criteria, you only include those with GDM history in your study. It is a confusing statement and please provide your justification.

RESPONSE: To address these comments, we have added the other part of the binary categories to Table 1.

We reported whether all of the participants were employed full time, part time, or were not employed (home parent). For those who were employed, we reported whether they were currently working or on maternity leave at the time of the interview. We have made this separation clearer in Table 1.

All of the participants had previously had GDM. Some had GDM in all of their pregnancies (or in their only pregnancy to date) while others had also had pregnancies where they did not have GDM.

Excerpt of Table 1:

|  | N (percent) |
| --- | --- |
| Ethnicity |  |
| White British or European | 14 (70) |
| Asian or Asian British | 6 (30) |
| **Chinese** | **2 (10)** |
| **Indian** | **3 (15)** |
| **Any other Asian background** | **1 (5)** |
| Employment **(when not on maternity leave)** |  |
| Full time | 10 (50) |
| Part time | 9 (45) |
| Home parent | 1 (5) |
| **On maternity leave** |  |
| **Yes** | **11 (58)** |
| **No** | **8 (42)** |
| **Household status** |  |
| Lives with partner | 18 (90) |
| **Does not live with partner** | **2 (10)** |
| **Number of pregnancies affected by GDM** |  |
| All pregnancies affected by GDM | 13 (65) |
| **Have also had normoglycaemic pregnancies** | **7 (35)** |
| **Management of GDM** |  |
| **Required medication (metformin and/or insulin)** | 10 (50) |
| **Managed by dietary and lifestyle changes alone** | **10 (50)** |

Table 1: You mentioned in the methodology (Line 111 – Line 113) that “ …. Participated in a pregnancy or GDM-related intervention or were considered unsuitable… were not invited”. Please explain why you include those on medication of GDM as depicted in Table 1. Do you accept those with medication of GDM in your study?

RESPONSE: We have clarified that women who “…participated in a pregnancy or GDM-related intervention **that was in addition to or in place of routine care (such as a** **clinical trial)**…” were not invited to take part in the methods (lines 139-140). Taking insulin and/or metformin during pregnancy is routine management of GDM, which we explained just above in the lines 136-137 of the methods.

Line 180: Please include the number of suggestion cards you have shown to the participants.

RESPONSE: We feel that we have clarified how many suggestion cards are referred to in this paper in earlier responses. In addition to the changes to the methods above, the cards are numbered (1–10) in Table 2 and each of the subheadings in the results.

Line 187: How do you define the agreement? What are the cut-offs for overall agreement, overall mixed response and overall disagreement? Do you have any references for the classification?

RESPONSE: As for the suggestion below, we feel that it is better to address this point in the methods rather than the table legend referred to in the line numbers. We have therefore added a paragraph in the methods to explain this:

“**To supplement the qualitative analysis, we classified the participants’ collective response to each suggestion card as overall agreement, disagreement or mixed in order to create a general indication or impression of their views. The classification was based on the authors’ interpretations of the participants’ responses: whilst we counted the number of agreements or disagreements, we also considered the vigour with which each participant responded. Where the classification was not obvious, consensus among the authors was sought.**” (lines 215-220).

We remind the readers of this by stating, “Overall agreement is based on the authors’ interpretation of the responses” in the legends of Table 2 and Table S3. To increase transparency, Table S3 indicates individuals’ responses on which the overall agreement was based.

Line 187: “… not all participants were shown each card, and some did not comment or agreement was unclear.”. Please justify why you exclude certain suggestion cards and how to define that “agreement was unclear”. This is important as it shows the quality of each suggestion card that you have proposed in your study.

RESPONSE: We have added additional descriptions to the methods to explain this:

“**Occasionally the interviewer decided not to show a particular participant a suggestion card if she considered that it was not appropriate given the earlier content of the interview and sometimes the participant did not provide clear feedback (e.g. due to natural distractions or initiating a different train of thought)**.” (lines 177-180).

We would like to emphasise that these suggestion cards were designed to prompt ideas and stimulate conversation (line 153) in these *semi-structured* interviews, just as the interview schedule is a guide rather than a prescriptive survey or structured interview.^1^

Information and understanding

Line 193 – Line 194 and Line 200: “… most of the participants”; “some would add…” and “… others had poor awareness” and “since most already had general…”. Please provide the actual number of participants.

Improving diet

Line 203 and Line 206 – Line 207: “The majority of the …”; “A couple of participants…” and “Others wanted advice that was relevant…”. Please provide the actual number of participants.

Improving physical activity

Line 215, Line 217, Line 221, Line 224, Line 226 and Line 231: “… Although many participants…”; “Some preferred…”; “… a few...”; “Almost all the participants..” and “Several participants….”: Please provide the actual number of participants.

Family

Line 237, Line 241, Line 243 and line 245: “…. Others walked with …..”; Some also found ….”; “Some participant reasoned that….”; “Others already knew …..”; “those that agreed wanted ….”: Please provide the actual number of participants.

Money

Line 252 and Line 253: “other participants considered …..”; “Some noted that cooking…”: Please provide the actual number of participants.

Monitoring

Line 256 and Line 262: “Almost all of the participants ….”; “at the same time, several were cautions ….”: Please provide the actual number of participants.

Sustainability

Line 266: “The majority of the participants ….”: Please provide the actual number of participants.

RESPONSE: We acknowledge that there are different views in qualitative analysis and reporting regarding quantification of the findings: some authors report participant frequencies as requested above whereas others consider that all quantification or even semi-quantification (many, most, etc.) should be avoided^11^ because we are not trying to ‘count’ experiences but instead describe the range of experiences and common themes. Reviewer #5 said, “As far as qualitative research is concerned, descripting in number is not recommended.”

In this paper, we have largely followed the approach suggested in an editorial note for the journal Addiction.^11^ We tend not to use terms that have a specific quantitative meaning (e.g. majority, minority, most) unless this is undoubtedly evident in the data. Instead, we more often report semi-quantification to support pattern recognition (“to draw attention to regularities, peculiarities and idiosyncrasies in the data”).^11^

What about others who did not want advice about sustaining changes? Please give more details on this.

RESPONSE: The participants who did not want more advice about sustaining changes were those who were not attempting to live healthier lifestyles themselves, which is logical. We have included this information in the text:

“**With the exception of those who were not attempting to eat a healthier diet and exercise more**, the participants wanted advice about sustaining changes and knew that maintaining a healthy lifestyle would be challenging.” (lines 337-338)

Delivery of support or intervention

Line 272: “The participants also suggested ….”: How many of them give additional suggestions?

Appointment with healthcare professionals

Line 283 and Line 293: “Most participants were keen ……”; “Many suggested that …..”: Please provide the actual number of participants.

Written information

Line 300: “The participants thought that ……”: How many participants?

RESPONSE: Please see the response above (page 13).

8. Discussion

Line 316: “… would help them to reduce their risk”: Please add “T2D” after the “risk”.

RESPONSE: We have added ‘of T2DM’:

“…would most help them to reduce their risk **of T2DM**”. (lines 388-389)

Line 317: “Many wanted more specific information about their long-term ……..”: Please add brief details of the specific information requested by the participants.

RESPONSE: We have added brief details about this:

“Many wanted more **individualised** information about their long-term risk **of T2DM after GDM, and how they might mitigate that risk,** but they often knew enough about the overall benefits of a healthy lifestyle.” (lines 389-391)

Line 323 – Line 324: “…. exercise between the review and this interview study”: Please give specific findings related to the previous review. Please add the reference of the review.

RESPONSE: The remainder of this paragraph describes how specific findings related to the previous review. We have made a few changes to help make this clearer:

“The DAiSIeS study was designed to build on our recent qualitative synthesis [17], bridging the gap between barriers and facilitators to diabetes prevention behaviours and intervention programme design. **Comparing the findings of that review [17] and this interview study,** we found that influences on healthy diet and exercise were similar, such as spending time with children instead of exercising and how the family could facilitate healthy behaviours. **We had reported** a lack of time and energy **as barriers to healthy lifestyles; this was also true in this interview study,** particularly in the early postpartum period that was considered to be a time for learning to adapt to life with their new baby. Women were more supportive of integrating activity into their daily routine than of participating in family-based exercise activities, which **had** appeared to be important in the review. Even though most of the DAiSIeS participants had a positive experience of GDM pregnancy and knew about having a healthy lifestyle, many felt that more specific information about lifestyle behaviours in T2DM risk prevention was important **(such as what foods would be best for them to eat)**. This echoes a participant in Lindmark *et al.* 2010 who said ‘…even if it is old knowledge it is good to hear it once more’ [26]. The participants tended only to maintain selected elements of **their** GDM diet**, which aimed to minimise spikes of high blood glucose during pregnancy,** because they considered it was too extreme to sustain **(such as a slight/moderate reduction in carbohydrate intake and strict avoidance of all high glycaemic index foods and high sugar fruit). Educational interventions** may therefore support them to learn what things to continue and what not to **in order to lose weight and maintain a balanced diet**. Of particular note, these changes were anticipated help women maintain healthy lifestyle in the long term.” (lines 394-414).

Line 326 – Line 327: “Lack of time and energy………, which was considered to be a time ……. new baby.”: Sentence unclear and do you have any reference to support this sentence? Please provide a reference to support this sentence.

RESPONSE: We have revised the sentence to read:

“**We had reported** a lack of time and energy **as barriers to healthy lifestyles; this was also true in this interview study,** particularly in the early postpartum period that was considered to be a time for learning to adapt to life with their new baby.” (lines 399-401).

It is not appropriate to provide a reference for this sentence as we are discussing the findings of this study.

Line 331: “…… many felt that more specific information about lifestyle……”: I believe that participants did provide specific information that they want to know more in the qualitative interview. Please add in the what are the specific information requested by the participants.

RESPONSE: We have provided an example of the information about lifestyle behaviours that the participants would like to know more about. We would also like to highlight that we cannot repeat all of the results in the discussion and that this was quite a top-level/general point.

“Even though most of the DAiSIeS participants had a positive experience of GDM pregnancy and knew about having a healthy lifestyle, many felt that more specific information about lifestyle behaviours in T2DM risk prevention was important **(such as what foods would be best for them to eat)**.” (lines 403-406).

Line 334: “….. tended only to maintain selected elements of the GDM diet because…”: Please elaborate on the elements or example of the GDM diet so that readers know how does GDM diet looks like. If GDM diet is difficult to sustain, then what mothers can do? Any other diet that is suitable for the mothers to tackle T2D?

RESPONSE: We have expanded on this sentence:

“The participants tended only to maintain selected elements of **their** GDM diet**, which aimed to minimise spikes of high blood glucose during pregnancy,** because they considered it was too extreme to sustain **(such as a slight/moderate reduction in carbohydrate intake and strict avoidance of all high glycaemic index foods and high sugar fruit). Educational interventions** may therefore support them to learn what things to continue and what not to **in order to lose weight and maintain a balanced diet**.” (lines 408-413).

Line 335: “Interventions may therefore support…”: Please suggest the type of intervention that will be beneficial to the participants.

RESPONSE: We have suggested that these might be educational interventions to support learning about how to lose weight and maintain a balanced diet, and report this statement in its fuller context here:

“…many felt that more specific information about lifestyle behaviours in T2DM risk prevention was important **(such as what foods would be best for them to eat)**. This echoes a participant in Lindmark *et al.* 2010 who said ‘…even if it is old knowledge it is good to hear it once more’ [26]. The participants tended only to maintain selected elements of **their** GDM diet**, which aimed to minimise spikes of high blood glucose during pregnancy,** because they considered it was too extreme to sustain **(such as a slight/moderate reduction in carbohydrate intake and strict avoidance of all high glycaemic index foods and high sugar fruit). Educational interventions** may therefore support them to learn what things to continue and what not to **in order to lose weight and maintain a balanced diet**. Of particular note, these changes were anticipated help women maintain healthy lifestyle in the long term.” (lines 405-413).

Line 336: “ …. these changes were anticipated help women ….”: The “changes” here refer to what kind of change? Please specify the type of change in your text.

RESPONSE: The sentence “Of particular note, these changes were anticipated help women maintain healthy lifestyle in the long term” concludes the paragraph in lines 394-413 (copied above) that describes the modifications to their lifestyles that women with GDM thought might help them to be healthier in the longer term. In context, we feel that this sentence is appropriate and it is clear that ‘the changes’ refer to what comes immediately before.

Line 338 – Line 339: Please briefly explain the previous findings of previous research.

RESPONSE: Some previous research has found that pregnancy is a suitable time for intervention, other research has found that postpartum is a suitable time. We have revised this to try to make it clearer:

“Previous studies have reported varying views regarding the **best** timing **for** intervention: **some suggest** during pregnancy [**17**,27,28] **while others suggest** postpartum [**17**–19].” (lines 415-417).

Line 340: “for more specific follow-up interventions during their pregnancy…:” Please suggest a suitable specific follow-up intervention in your conclusion.

RESPONSE: After describing *what* interventions might be helpful to women with a history with GDM in lines 394-413 (copied above), we move on to discussing the timing and delivery of them in the following paragraph (lines 413-418). We have clarified this by saying:

“Previous studies have reported varying views regarding the **best** timing **for** intervention: **some suggest** during pregnancy [**17**,27,28] **while others suggest** postpartum [**17**–19]. We concluded that women with GDM should be prepared for more specific follow-up interventions **such as those described above** during their pregnancy, provided that this is done in a sensitive manner, echoing the findings of Ingstrup *et al.* regarding the importance of rapport with peer councillors [29].” (lines 415-420).

9. Strength and limitations

Line 357: “yet we were unable …… for support”: Do you think future study needs to add in BMI data in the interview to elucidate the influence of BMI towards the requirement for support? If yes, please add a sentence on this.

RESPONSE: We think that the key point is that the advice and support is important regardless of BMI. We have made a small adjustment to this section to emphasise this:

“We did not collect data on BMI or whether participants were overweight because we did not access their medical record, and to ask them to self-report this could be insensitive during the postpartum period. It is important **to facilitate** all women to maintain a healthy diet and be physically active after GDM, regardless of their BMI, yet we were unable to comment on whether BMI affected requirements for support.” (lines 436-440).

10. Implications for practice

Line 372: “support the important role clinicians play…”: Please add “of” before “clinicians”.

RESPONSE: We have added ‘that’ to this sentence (adding “of” before “clinicians” would not make sense):

“Our findings support the important role **that** clinicians play in promoting healthy behaviours and signposting resources during pregnancy and postpartum [35].” (lines 455-456).

11. Supporting information

Table S3: How do you define the agreement? What are the cut-offs for overall agreement, overall mixed response and overall disagreement? Do you have any references for the classification?

RESPONSE: We have added this description to the methods (lines 215-220), as explained on page 12 of this response letter.

Reviewer **#5**

In the methods section, the trustworthiness of the data was not indicated.

RESPONSE: In qualitative research, trustworthiness most often refers to Lincoln and Guba’s criteria of credibility, transferability, dependability, and confirmability.^12^ The following descriptions and strategies are taken from Korstjensa and Moser 2018, Boxes 1 and 2.^12^

- Credibility, ‘the confidence that can be placed in the truth of the research findings,’ can be ensured by *investigator triangulation* (where two or more researchers to make coding, analysis and interpretation decisions) and *member check* (feeding back data, analytical categories, interpretations and conclusions to members of those groups from whom the data were originally obtained). We describe how we have used these approaches in the methods:

Investigator triangulation: “The first author coded all of the transcripts and developed the charts, and another author coded and charted four transcripts to ensure **general** agreement **with the coding strategy and discuss alternative explanations.** The other authors read some or all of the transcripts and charts in order to support interpretation. During these discussions, we considered the authors’ clinical (obstetrics, diabetes and general practice) and non-clinical backgrounds **and made notes to record the analytical and interpretational decisions**.” (lines 209-214).

Member check: “We also invited the participants to provide feedback on a summary of the findings (not the transcripts) and incorporated any responses into the final version.” (lines 221–222).

- Transferability, ‘the degree to which the results of qualitative research can be transferred to other contexts or settings with other respondents. The researcher facilitates the transferability judgment to a potential user through *thick description*’ (describing not just the behaviour and experiences, but their context as well, so that the behaviour and experiences become meaningful to an outsider). We have done this throughout the paper, for example:
  - Describing the general experiences of women with a history of GDM in the introduction (lines 80-91),
  - Describing how GDM is diagnosed and managed at the hospitals the participants attended in the methods (lines 126-137),
  - The results begin with a general description of our participants’ views and experiences of GDM (lines 234-237) and postpartum regarding a healthy lifestyle (lines 238-247),
  - Reporting each of the themes with thick description: e.g. “Improving physical activity (suggestion cards 5 and 7). Although many participants reported doing less physical activity than before pregnancy, several prioritised running (while their partner looked after the children), dance classes or home workouts. Others did lower-intensity activity, like pushing the buggy. Many wanted to do more exercise, and felt this would be achievable when the children were older, they finished breastfeeding, or had better recovered from pregnancy [the context]. Help to exercise with others was frequently considered to facilitate physical activity because… [the specific finding].” (lines 285-291).
- Dependability and confirmability, involving ‘participants’ evaluation of the findings, interpretation and recommendations of the study such that all are supported by the data as received from participants of the study’ and ‘the degree to which the findings of the research study could be confirmed by other researchers… the findings are not figments of the inquirer’s imagination, but clearly derived from the data’. Korstjensa and Moser explain that both dependability and confirmability can be confirmed by an *audit trail* (transparently describing the research steps taken from the start of a research project to the development and reporting of the findings, and keeping records of the research path).

Audit trail: We have used framework analysis in line with accepted standards and have documented the approach (e.g. lines 189-214 of the methods) and include the framework itself in S2 Table. We also report that field notes were made after each interview (“**The interviewer** recorded **reflexive field notes** on each interview **including the context (e.g. setting, if anyone else was nearby) and subjective reflections**” [lines 184-185]) and have added that “During these discussions, we… **made notes to record the analytical and interpretational decisions**.” (lines 212-214).

- Reflexivity, ‘the process of critical self-reflection about oneself as researcher… and the research relationship’, should be recorded and examined using a *diary*.

Diary: We have explained that “The interviewer recorded **reflexive field notes** on each interview **including the context (e.g. setting, if anyone else was nearby) and subjective reflections**” (lines 184-185) and in the analysis that “The other authors read some or all of the transcripts and charts in order to support interpretation. During these discussions, we considered the authors’ clinical (obstetrics, diabetes and general practice) and non-clinical backgrounds **and made notes to record the analytical and interpretational decisions**.” (lines 211-214).

Data collection technique analysis and report were not meticulously stated. The author should have used COREQ.

RESPONSE: We have included the COREQ checklist as part of the submission, and we understand that it is available for the reviewers to download.

We have expanded on the analysis and reporting in response to the reviewers above and double-checked that all elements of the COREQ checklist are included within the paper.

The authors have not clearly stated how the themes emerged. They have used a card which is pre-specified and may not explore the phenomenon very well.

RESPONSE: We conducted a framework analysis in line with the methodological publications and relevant reporting guidelines. Additionally, the framework is included in Table S2. We have added more details and restructured the analysis section of the methods:

“We used a framework approach to analyse the interviews [25], with the aid of NVivo 12 (QSR International Pty Ltd; version 12; released 2018):

1. **Familiarisation:** We familiarised ourselves with **the data by listening to** the recordings **and reading the** transcripts and field notes, **and making notes about important concepts**.
2. **Identifying a thematic framework: We** developed a thematic framework that was based on the suggestion cards then refined **it** **as required upon analysing the interviews by incorporating additional repeating concepts (such as communication requires a positive, non-judgemental tone).** We distinguished between suggestions initiated by participants and responses to the suggestion cards in the analysis. The final codebook for the framework is reported in S2 Table.
3. **Indexing:** Next, we coded each transcript **according to the thematic framework**.
4. **Charting: We** drew charts to summarise **what each participant said in relation to each part of the thematic framework. One row was used for each participant interviewed, and one column was used for each code within the framework.**
5. **Mapping and interpretation:** We carefully studied the charts for repeating or **characteristic** ideas to describe and explain the phenomena observed. **Where differences and deviant cases were observed, we attempted to understand the ways in which they different and why, according to the information the participants provided.**

The first author coded all of the transcripts and developed the charts, and another author coded and charted four transcripts to ensure **general** agreement **with the coding strategy and discuss alternative explanations.** The other authors read some or all of the transcripts and charts in order to support interpretation. During these discussions, we considered the authors’ clinical (obstetrics, diabetes and general practice) and non-clinical backgrounds **and made notes to record the analytical and interpretational decisions**.” (lines 189-214).

As explained in lines 150-151 and Fig 1, the suggestion cards were based on the findings of a systematic review on a very similar research question. The suggestion cards were only used as prompts (line 153) to give the participants ideas about what kinds of interventions might be available. Additionally, we have clarified that:

“Participants were asked about their own ideas [for support] first, then to comment on the **ten** suggestion cards provided by the interviewer (e.g. whether they agreed, disagreed or would add anything). **It was emphasised that disagreement (they probably would not find that intervention helpful) as equally useful to hear as agreement (they might find that intervention helpful).** If the participants did not want to make any further changes themselves, they were asked what they thought might help others with GDM based on their own experience.” (lines 171-176).

This is repeated in the discussion: “Furthermore, social desirability bias may have led some participants to agree with the prompted suggestions, although it was clarified in the question that negative responses would be as informative as positive ones.” (lines 440-442).

The quotations used in your manuscript contain potentially identifying information. Please amend your manuscript by either limiting the amount of potentially-identifying information presented, or by obtaining explicit consent to publish such information. To limit the identifying information, please remove the ages and occupation information from your quotes to help maintain participant confidentiality. Please use age ranges in place of the ages. Please check that the identifiers do not link to participants.

RESPONSE: We have reviewed each of the quotes included in the manuscript and confirm that they *do not* contain potentially identifying information. At no point do we report individuals’ ages and occupation information; we only collected their age range and employment status e.g. as part or full time.

As far as qualitative research is concerned, descripting in number is not recommended. However, the authors stated state the number of women who were aware of the link between GDM and T2D, which would significantly affect the interpretation of the results.

RESPONSE: We report that “…and two participants explained that they had been unaware of an association between GDM and T2DM” (lines 245-246). This was unexpected because informing women with GDM about their future risk of T2D is usually part of GDM pregnancy care in this region. Additionally, we stated this in the Participant Information Sheet in the justification for the study. Where it came up as ‘new’ information during the interview, we directed women to the Gestational Diabetes UK website (www.gestationaldiabetes.co.uk) or suggested that they contact their GP. We also provided a link to this website in the summary of the findings sent to the participants at the end of the study.

The fact that women with GDM have had opportunities to learn this information during pregnancy but have not taken it in or remembered it afterwards highlights we importance of effective communication at such an overwhelming time and then postpartum. This is considered in the first theme of the results (Information and understanding).

Generally, the title is of interest and current issue.

The paper can be accepted for publication after modification.

RESPONSE: We would like to thank the reviewer for their comments on our paper.

**Reviewer #6 (attached comments)**

Title - There is a tautology in the title (A qualitative interview study exploring how to support a healthy diet and physical activity after gestational diabetes). The second part of the title is more suitable and straightforward than (“post-GDM support would be really good for mothers”: a qualitative interview study exploring how to support a healthy diet and physical activity after gestational diabetes). The title's preamble and the title itself are the same, so the title should be reframed to make more coincide.

RESPONSE: We would like to keep the title as *“Post-GDM support would be really good for mothers”: a qualitative interview study exploring how to support a healthy diet and physical activity after gestational diabetes*, unless the editor has strong preferences. The first part is a quote from a participant that highlights women with GDM’s desire for support, therefore is not redundant.

It also follows the pattern of our other paper on these interviews, as well as others. For example:

- “Oh, I've got an appointment”: A qualitative interview study exploring how to support attendance at diabetes screening after gestational diabetes
- “Acknowledge me as a capable person”: How people with mental ill health describe their experiences with general emergency care staff - A qualitative interview study
- “The office of disaster management” Nurse managers' experiences during COVID-19: A qualitative interview study using thematic analysis, and
- “Whole life changed” - Experiences of how symptoms derived from acute pulmonary embolism affects life. A qualitative interview study”).

**Abstract**

Line 24 - developing type 2 diabetes mellitus (T2DM). Be specific about the type of diabetes being discussed.

RESPONSE: We have replaced T2D with T2DM throughout.

Lines 26/27 - The research sought to explore women’s views on suggested … maybe more appropriate

RESPONSE: We have changed from the active to passive voice throughout the abstract (as described specifically below), but are happy to revert these changes if the editor prefers.

Firstly, we have changed the aim from “We sought to explore women’s views on…” to “**This study** sought to explore women’s views on…” (line 26).

Lines 28/29 - Semi-structured interviews with 20 participants in Cambridgeshire, UK at 3–48 months after GDM were conducted.

RESPONSE: We have changed the first sentence of the Methods from “We conducted semi-structured interviews with 20 participants in Cambridgeshire, UK …” to “Semi-structured interviews with 20 participants in Cambridgeshire, UK **were conducted**…” (lines 29-30).

Lines 29/30 - ~~Their~~ current diet and physical activity, intentions for any changes, and views on potential interventions to help manage T2DM risk through these behaviours were discussed.

RESPONSE: We have changed this sentence from “Their current diet and physical activity, intentions for any changes, and views on potential interventions to…” to “**The participants’** current diet and physical activity, intentions for any changes, and views on potential interventions to…” (line 30).

Lines 31/32 - ~~We based~~ the interview schedule, suggested interventions, and thematic framework was based on a recent systematic review

RESPONSE: We have changed this sentence from “We based the interview schedule, suggested interventions, and thematic framework on a recent systematic review” to “The interview schedule, suggested interventions, and thematic framework **were** **based** on a recent systematic review” (line 33).

Lines 53/54 - which percentage is the emphasis on? The bracket?

RESPONSE: We have clarified that the brackets are the 95% confidence intervals for these statistics:

“… development of T2DM is 18% (**95% confidence interval** 5–34%) higher per unit BMI at follow-up, and 57% (**95% confidence interval** 39–70%) lower in White European populations compared to other populations (adjusting for ethnicity and follow-up) [7]. (lines 62-65).

**Introduction**

Lines 65/73 - The context in this session is in isolation.

RESPONSE: In order to link this paragraph with the one above, we have amended the first sentence to read:

“**In order to understand the facilitators and barriers towards lifestyle in women with a history of GDM, we conducted a qualitative synthesis of their views on reducing their risk of developing diabetes postpartum through lifestyle and behaviour changes [17]. We** found that women who had had GDM…” (lines 80-83).

Additionally, after explaining some of the findings of that paper, we link this to our aim by saying:

“**There is therefore a gap in recent literature in the UK population surrounding the acceptability of recommendations for intervention after GDM in a real-life context.** In this study, **we sought to address this gap by exploring** the views of women with a history of GDM on possible interventions to support healthy diet and physical activity to reduce diabetes risk, in addition to participants’ own suggestions. We aimed to identify the most promising interventions for future development.” (lines 92-97).

**Participants and Methods**

Recruitment: this session sounds more like a narration due to the use of "we."

RESPONSE: Use of the active voice in common practice in the methods section in publications published in PLoS ONE (for example^13–15^). We would be happy to amend this if the editor feels that would be better, however.

Lines 118-120 - Interview process: informed consent implies “they understood the purpose and procedure of the study and that they could stop the interview at any point, and were happy for it to be audio-recorded”. Hence, this addition may not be necessary.

RESPONSE: We agree with the reviewer’s point. However, the Critical Appraisal Skills Programme (CASP)^16^ checklist for appraising qualitative research states the following:

‘7. Have ethical issues been taken into consideration? Consider • If there are sufficient details of how the research was explained to participants for the reader to assess whether ethical standards were maintained; • If the researcher has discussed issues raised by the study (e.g. issues around informed consent or confidentiality or how they have handled the effects of the study on the participants during and after the study…)’

We therefore wanted to include more details relating to the ethical considerations and consent.

Lines 89/90 - Why is the reference?

RESPONSE: Please see the response above – we have revised this to “**As widely recommended for data saturation [21]**, we finished recruitment after several interviews did not lead to novel findings”. (lines 116-117).

**Results**

Well presented

RESPONSE: Thank you for this feedback.

**Conclusion**

Lines 406 - The range of formats could be mentioned.

RESPONSE: We have revised this to read:

“This support could be provided throughout pregnancy and postpartum, in a range of formats **including face-to-face with healthcare providers or peers and online or physical written information**.” (lines 491-493).

**References**

Well presented.

________________________________________

7. PLOS authors have the option to publish the peer review history of their article (what does this mean?). If published, this will include your full peer review and any attached files.

If you choose “no”, your identity will remain anonymous but your review may still be made public.

Do you want your identity to be public for this peer review? For information about this choice, including consent withdrawal, please see our Privacy Policy.

Reviewer #3: No

Reviewer #4: No

Reviewer #5: No

Reviewer #6: No

**Journal Requirements**

When submitting your revision, we need you to address these additional requirements.

1. Please ensure that your manuscript meets PLOS ONE's style requirements, including those for file naming. The PLOS ONE style templates can be found at https://journals.plos.org/plosone/s/file?id=wjVg/PLOSOne_formatting_sample_main_body.pdf and https://journals.plos.org/plosone/s/file?id=ba62/PLOSOne_formatting_sample_title_authors_affiliations.pdf

RESPONSE: We have checked that the manuscript meets these style requirements.

2. We note that the grant information you provided in the ‘Funding Information’ and ‘Financial Disclosure’ sections do not match. When you resubmit, please ensure that you provide the correct grant numbers for the awards you received for your study in the ‘Funding Information’ section.

RESPONSE: We have ensured that the Funding Information and Financial Disclosure are consistent.

3. We note that you have indicated that data from this study are available upon request. PLOS only allows data to be available upon request if there are legal or ethical restrictions on sharing data publicly. For information on unacceptable data access restrictions, please see <http://journals.plos.org/plosone/s/data-availability#loc-unacceptable-data-access-restrictions>.

In your revised cover letter, please address the following prompts:

a) If there are ethical or legal restrictions on sharing a de-identified data set, please explain them in detail (e.g., data contain potentially identifying or sensitive patient information) and who has imposed them (e.g., an ethics committee). Please also provide contact information for a data access committee, ethics committee, or other institutional body to which data requests may be sent.

b) If there are no restrictions, please upload the minimal anonymized data set necessary to replicate your study findings as either Supporting Information files or to a stable, public repository and provide us with the relevant URLs, DOIs, or accession numbers. Please see http://www.bmj.com/content/340/bmj.c181.long for guidelines on how to de-identify and prepare clinical data for publication. For a list of acceptable repositories, please see http://journals.plos.org/plosone/s/data-availability#loc-recommended-repositories.

We will update your Data Availability statement on your behalf to reflect the information you provide.

RESPONSE: The data underlying the findings of this study are qualitative data (interview transcripts).

PLOS’ *Guidelines for qualitative data* (http://journals.plos.org/plosone/s/data-availability) state:

“For studies analyzing data collected as part of qualitative research, authors should make excerpts of the transcripts relevant to the study available in an appropriate data repository, within the paper, or upon request if they cannot be shared publicly. If even sharing excerpts would violate the agreement to which the participants consented, authors should explain this restriction and what data they are able to share in their Data Availability Statement.”

As requested, we have made excerpts of the transcripts relevant to the study available within the paper and upon request. Three of the four reviewers confirm that data availability is acceptable in question 4 of their feedback.

We have ethical approval and consent from participants to share the pseudo-anonymised transcripts with researchers upon their request to us directly, via the corresponding author’s contact details, but not to make them publicly available. As we have reported in the manuscript, the study was approved by West London and GTAC Research Ethics Committee (reference 19/LO/0441).

This is in line with the data availability statements of other qualitative studies published in PLoS ONE in 2021 such as Backhausen *et al.* or Fleming *et al.*^13,17^

**References**

1.	DeJonckheere M, Vaughn LM. Semistructured interviewing in primary care research: A balance of relationship and rigour. *Fam Med Community Heal*. 2019;7(2):e000057. doi:10.1136/fmch-2018-000057

2.	Dennison RA, Ward RJ, Griffin SJ, Usher-Smith JA. Women’s views on lifestyle changes to reduce the risk of developing Type 2 diabetes after gestational diabetes: a systematic review, qualitative synthesis and recommendations for practice. *Diabet Med*. 2019;36(6):702-717. doi:10.1111/dme.13926

3.	Meek CL, Lindsay RS, Scott EM, et al. Approaches to screening for hyperglycaemia in pregnant women during and after the COVID-19 pandemic. *Diabet Med*. 2021;38(1):doi: 10.1111/dme.14380

4.	Lachmann EH, Fox RA, Dennison RA, Usher-Smith JA, Meek CL, Aiken CE. Barriers to completing oral glucose tolerance testing in women at risk of gestational diabetes. *Diabet Med*. 2020;37(9):1482-1489. doi:10.1111/dme.14292

5.	Dworkin SL. Sample Size Policy for Qualitative Studies Using In-Depth Interviews. *Arch Sex Behav*. 2012;41(6):1319-1320. doi:10.1007/s10508-012-0016-6

6.	Ezzy D. Are qualitative methods misunderstood? *Aust N Z J Public Health*. 2001;25(4):294-297. doi:10.1111/j.1467-842X.2001.tb00582.x

7.	Galdas P. Revisiting Bias in Qualitative Research: Reflections on Its Relationship With Funding and Impact. *Int J Qual Methods*. 2017;16(1). doi:10.1177/1609406917748992

8.	Dennison RA, Fox RA, Ward RJ, Griffin SJ, Usher-Smith JA. Women’s views on screening for Type 2 diabetes after gestational diabetes: a systematic review, qualitative synthesis and recommendations for increasing uptake. *Diabet Med*. 2020;37(1):29-43. doi:10.1111/dme.14081

9.	Dennison RA, Meek CL, Usher- JA, et al. ‘Oh, I’ve got an appointment’: A qualitative interview study exploring how to support attendance at diabetes screening after gestational diabetes. *Diabet Med*. 2021;00:e14650. doi:10.1111/dme.14650

10.	Barbour RS. Checklists for improving rigour in qualitative research: a case of the tail wagging the dog? *BMJ*. 2001;322(7294):1115-1117. doi:10.1136/bmj.322.7294.1115

11.	Neale J, Miller P, West R. Reporting quantitative information in qualitative research: Guidance for authors and reviewers. *Addiction*. 2014;109(2):175-176. doi:10.1111/add.12408

12.	Korstjens I, Moser A. Series: Practical guidance to qualitative research. Part 4: Trustworthiness and publishing. *Eur J Gen Pract*. 2018;24(1):120-124. doi:10.1080/13814788.2017.1375092

13.	Backhausen MG, Iversen ML, Sköld MB, Thomsen TG, Begtrup LM. Experiences managing pregnant hospital staff members using an active management policy—A qualitative study. Doraiswamy S, ed. *PLoS One*. 2021;16(2):e0247547. doi:10.1371/journal.pone.0247547

14.	Stamov Roßnagel C, Lo Baido K, Fitzallen N. Revisiting the relationship between constructive alignment and learning approaches: A perceived alignment perspective. Zhou M, ed. *PLoS One*. 2021;16(8):e0253949. doi:10.1371/journal.pone.0253949

15.	Galarneau LR, Hilburt J, O’Neill ZR, et al. Experiences of people with opioid use disorder during the COVID-19 pandemic: A qualitative study. Leong C, ed. *PLoS One*. 2021;16(7):e0255396. doi:10.1371/journal.pone.0255396

16.	Critical Appraisal Skills Programme (Qualitative Research Checklist). Available from: https://casp-uk.net/casp-tools-checklists. Date last accessed: 01 Aug 2021.

17.	Fleming T, Collins AB, Bardwell G, et al. A qualitative investigation of HIV treatment dispensing models and impacts on adherence among people living with HIV who use drugs. Ahmed SI, ed. *PLoS One*. 2021;16(2):e0246999. doi:10.1371/journal.pone.0246999
